# Editorial: User-Avatar Bond: Risk and Opportunities in Gaming and Beyond

**DOI:** 10.3389/fpsyg.2022.923146

**Published:** 2022-05-23

**Authors:** Vasileios Stavropoulos, Rabindra Ratan, Kwan Min Lee

**Affiliations:** ^1^Institute for Health and Sport, Victoria University, Footscray, VIC, Australia; ^2^Department of Media and Information, Michigan State University, East Lansing, MI, United States; ^3^User-Experience Lab, Nanyang Technological University, Singapore, Singapore

**Keywords:** user-avatar bond, virtual reality, gaming, identification, embodiment

The proliferation and advancement of gaming and social media applications with embedded virtual reality features, such as virtual worlds and avatars, have significantly enhanced the potential for these media technologies to attract and engage users, alongside broadening usage and popularity (Stavropoulos et al., 2021). Nevertheless, concerns have also emerged about the perils to well-being arising from excessive usage and addiction-like behaviors (Colder Carras et al., 2021). Along this line, research continues to examine how the use of virtual reality enriched, and/or gamified applications, may either enhance or undermine one's wellbeing (Kruzan and Won, 2019; Stavropoulos et al., 2020; Houwelingen-Snippe et al., 2021). To respond to such questions and interpret users' adaptive and/or maladaptive trajectories, scholars aim to demystify the interplay between influences related to the user, their real-life surroundings, and the application itself (Stavropoulos et al., 2021; Lee and Lee, 2022; Ratan et al., 2022). Within this context, the avatar, or self-representation of the human user that facilitates engagement in a mediated environment (Nowak and Fox, 2018), has captivated scholar interest. While the term “avatar” originated from the Sanskrit “avatāra”, meaning the re-incarnation of Hindu spirits in terrestrial form (Bailenson and Blascovich, 2004), it has become a central element of modern virtual experiences in which users control virtual entities resembling themselves in physical, behavioral, and/or psychological ways (Lee, 2004). Contributing to this line of inquiry as editors of a *Frontiers* Research Topic, we have assembled a collection of studies focused on the notion of the user-avatar bond (UAB).

The UAB relates to multiple previous areas of avatar-related research. Their many theoretical constructs have been developed to describe users' psychological connections with their avatars, such as embodiment (Kilteni et al., 2012; Peck and Gonzalez-Franco, 2021), monadic identification (Klimmt et al., 2010), polythetic identification (Downs et al., 2019), self-presence (Biocca, 1997; Ratan, 2012), avatar-self relevance (Ratan and Dawson, 2016), and the player-avatar relationship typology (Banks, 2015). Although distinct in name, these constructs often contain overlapping concepts, such as embodiment as a facet of avatar identification (Van Looy et al., 2012). As such, for this Research Topic we use the umbrella concept of UAB to include any research that involves any notions of a psychological association between the user and avatar, allowing us to focus on the notable correlates of such inter-connections. For example, multiple studies have found that when users customize their avatars, they experience an increased sense of closeness with them, which enhances their identification, enjoyment, and other outcomes of use (Hefner et al., 2007; Trepte and Reinecke, 2010; Birk et al., 2016; Kang and Kim, 2020; Koulouris et al., 2020). Further, sense of embodiment likely plays a role in the Proteus effect, of the phenomenon that an avatar's characteristics influence the user's subsequent behaviors (Yee and Bailenson, 2007, 2009; Ratan et al., 2020). Thus, while the individual's real-life background and knowledge, either directly and/or indirectly, predicate the avatar's formation, the avatar can also influence the user's conduct outside of the virtual world in subtle, although significant ways (e.g., distorted perceptions, cognitions, and even non-deliberate actions Ortiz de Gortari and Gackenbach, 2021; Navarro et al., 2022).

Research on the UAB is essential to developing an understanding of how avatar use may impact individual users specifically, as well as on the broad scale. In that extent, the UAB has been associated with both negative and positive outcomes, varying from disordered gaming vulnerability to improvements in e-health and cyber-education applications (Mastorci et al., 2020; Mkrttchian et al., 2020; Stavropoulos et al., 2020; Szolin et al., 2022). For instance, tendencies to identify with, embody, and even idealize the avatar potentially underpin the ability for games to help users battle depression and anxiety or develop dexterities necessary for professional performance (Peña et al., 2020). Ironically, the same engaging properties have been proposed to explain the UAB's contribution to disordered gaming patterns (Stavropoulos et al., 2019, 2020).

To address this complex dynamic, the present Research Topic includes a set of 14 studies coming from widely different, though contemporaneously complimentary perspectives. These articles capture recent global advancements in the field, deriving from Singapore (Chen et al.), the USA (Alon et al.; Banks and Bowman; Huang-Isherwood and Peña; Jeong et al.; Prettyman and Bolls; Wang et al.; Lynch et al.), Korea (Ahn et al.; Erb et al.), Canada (Czerwonka et al.) and Taiwan (Lin and Wu; Lin et al.). Furthermore, the studies informing this Research Topic reflect the current methodological diversity embraced by leading UAB scholars, including model-driven literature reviews (Lynch et al., 2022), thematic analysis (Erb et al.) and a series of advanced experimental and quantitative designs (Alon et al.; Chen et al.; Lin and Wu; Lin et al.; Prettyman and Bolls). Subsequently, the range of analytical protocols operationalizing the various methods employed, include the parallel assessment of physical and virtual kinematics (Jeong et al.), semantic network analysis (Banks and Bowman), ordinary least squares (OLS) regressions (Wang et al.), analysis of variance and independent sample *t*-tests (Huang-Isherwood and Peña), mediation modeling (Ahn et al.), and a novel avatar-affordances framework analysis (Czerwonka et al.).

Such methodological and analytical wealth allows the results of the present Research Topic to significantly expand the available knowledge surrounding both (1) the conceptualization and the multifaceted description and (2) the applicability of the UAB. Indicatively, and considering the UAB concept, (1a) major underlying themes of “tolerance”, “malleability” and “flexibility” were revealed (Erb et al.), and (1b) a new skilled-driven theoretical scaffolding of UABs was introduced (Lynch et al., 2022). Considering means contributing to the multifaceted UAB description, (1c) a ground-breaking creative method blending physical and virtual kinematics to portray the bond was introduced (Jeong et al.), while (1d) universal and idiosyncratic player-avatar relationship patterns were differentiated (Banks and Bowman). Despite these, (1e) avatar customization options in exergames were found to limit players from presenting the way they may have liked in the virtual world (Czerwonka et al.), challenging UAB's independence from real-world existing social biases. Considering the practical usage of the UAB, promising application domains related to (2a) physical activity, (2b) morality/risky behaviors, (2c) sexual identity and (2d) minorities' acceptance emerged. In relation to physical activity in particular, (2a1) the utilization of cartoon-avatars, that resemble children's body type, was shown to enhance engagement with active video games, leading to an increase in physical activity motivation (Alon et al.). Relatedly, (2a2) participants' gender and age were found to influence the effect of avatar body depiction on exercise engagement (Lin et al.; Lin and Wu). Together, these studies suggest that the interaction of user and avatar identity characteristics play an important role in the Proteus effect, potentially through mechanisms of social (e.g., upward) comparison. Considering morality, (2b1) an avatar's perceived morals were found to effect players' guilt and attributional responses (Ahn et al.), while (2b2) the UAB was found to influence feelings of guilt after committing game-simulated violent acts (Huang-Isherwood and Peña). Similarly, (2b3) identification with avatars in a virtual dating game was associated with choosing less sexually risky behaviors (Wang et al.). Considering sexual identity, (2c1) more strongly sex-typed participants were found to be more motivated by higher sex-salient avatars, as reflected by skin conductance and facial electromyography assessments during gameplay (Prettyman and Bolls). Finally, considering minorities' acceptance, (2d1) outgroup-member avatar embodiment was found to effectively improve the reception of immigrants (Chen et al.).

Despite the significant knowledge attained and the progress made, obstacles in maximizing the benefits and minimizing the risks related to the employment of UAB persist. A vast proportion of researchers continue to approach UAB from either a positive and/or a negative point of view without addressing the continuum of adaptive-to-maladaptive applications that avatar use may accommodate (Stavropoulos et al., 2021). There is a scarcity of multilevel UAB studies, examining within- and between-individual differences, whilst taking into consideration customization and application effects, as Lynch et al. would propose. A variety of psychometric scales assessing UAB experiences have been employed, limiting inevitably the comparability of international findings. The employment of modern methodologies, such as network analysis in the context of semantics (Banks and Bowman) and the comparative study of real and virtual kinematics (Jeong et al.) remain rare. Finally, interdisciplinary collaborations between communication, health, and allied health UAB researchers are present only in a minority of studies.

In this context, our conclusion is 2-fold. First, irrespective of proclamations on the “positive” and/or “negative” nature of UAB, *more consistent measurement and conceptualization approaches surrounding the composition and structure of the UAB are needed*, so that future studies' findings may be adequately compared and integrate to inform practice. Secondly, we must recognize that the communication-related, psychological, and information-science, past and present advancements in the field of UAB will provide the basis for future research and practice in our society's impending *metaverse* era.

## Author Contributions

VS and RR contributed to the literature review, the structure, and sequence of theoretical arguments. KL contributed to the theoretical consolidation of the current work, revised, and edited the final manuscript. All authors contributed to the article and approved the submitted version.

## Funding

VS was funded by the Australian Research Council Discovery Early Career Researcher Award DE210101107.

## Conflict of Interest

The authors declare that the research was conducted in the absence of any commercial or financial relationships that could be construed as a potential conflict of interest.

## Publisher's Note

All claims expressed in this article are solely those of the authors and do not necessarily represent those of their affiliated organizations, or those of the publisher, the editors and the reviewers. Any product that may be evaluated in this article, or claim that may be made by its manufacturer, is not guaranteed or endorsed by the publisher.
